# Arterial and Venous Thrombosis Complicated in COVID-19: A Retrospective Single Center Analysis in Japan

**DOI:** 10.3389/fcvm.2021.767074

**Published:** 2021-11-19

**Authors:** Seiya Oba, Tadashi Hosoya, Miki Amamiya, Takahiro Mitsumura, Daisuke Kawata, Hirokazu Sasaki, Mari Kamiya, Akio Yamamoto, Takahiro Ando, Sho Shimada, Tsuyoshi Shirai, Tsukasa Okamoto, Tomoya Tateishi, Akira Endo, Junichi Aiboshi, Nobuyuki Nosaka, Hideo Yamanouchi, Toyomu Ugawa, Eiki Nagaoka, Keiji Oi, Susumu Tao, Yasuhiro Maejima, Yukie Tanaka, Kousuke Tanimoto, Hiroaki Takeuchi, Shuji Tohda, Akihiro Hirakawa, Tetsuo Sasano, Hirokuni Arai, Yasuhiro Otomo, Yasunari Miyazaki, Shinsuke Yasuda

**Affiliations:** ^1^Department of Rheumatology, Graduate School of Medical and Dental Sciences, Tokyo Medical and Dental University (TMDU), Tokyo, Japan; ^2^Department of Cardiovascular Medicine, Graduate School of Medical and Dental Sciences, Tokyo Medical and Dental University (TMDU), Tokyo, Japan; ^3^Department of Respiratory Medicine, Graduate School of Medical and Dental Sciences, Tokyo Medical and Dental University (TMDU), Tokyo, Japan; ^4^Trauma and Acute Critical Care Medical Center, Graduate School of Medical and Dental Sciences, Tokyo Medical and Dental University (TMDU), Tokyo, Japan; ^5^Department of Intensive Care Medicine, Graduate School of Medical and Dental Sciences, Tokyo Medical and Dental University (TMDU), Tokyo, Japan; ^6^Department of Cardiovascular Surgery, Graduate School of Medical and Dental Sciences, Tokyo Medical and Dental University (TMDU), Tokyo, Japan; ^7^Research Core, Institute of Research, Tokyo Medical and Dental University (TMDU), Tokyo, Japan; ^8^Genome Laboratory, Medical Research Institute, Tokyo Medical and Dental University (TMDU), Tokyo, Japan; ^9^Department of Molecular Virology, Tokyo Medical and Dental University (TMDU), Tokyo, Japan; ^10^Clinical Laboratory, Tokyo Medical and Dental University (TMDU) Hospital, Tokyo, Japan; ^11^Department of Clinical Biostatistics, Graduate School of Medical and Dental Sciences, Tokyo Medical and Dental University (TMDU), Tokyo, Japan

**Keywords:** thrombosis, COVID-19, coagulopathy, D-dimer, variant of concern

## Abstract

**Background:** Thrombosis is a characteristic complication in coronavirus disease 2019 (COVID-19). Since coagulopathy has been observed over the entire clinical course, thrombosis might be a clue to understanding the specific pathology in COVID-19. Currently, there is limited epidemiological data of COVID-19-associated thrombosis in the Japanese population and none regarding variant strains of SARS-CoV-2. Here, we elucidate the risk factors and the pattern of thrombosis in COVID-19 patients.

**Methods:** The patients consecutively admitted to Tokyo Medical and Dental University Hospital with COVID-19 were retrospectively analyzed. SARS-CoV-2 variants of concern/interest (VOC/VOI) carrying the spike protein mutants E484K, N501Y, or L452R were identified by PCR-based analysis. All thrombotic events were diagnosed by clinical symptoms, ultrasonography, and/or radiological tests.

**Results:** Among the 516 patients, 32 patients experienced 42 thromboembolic events. Advanced age, severe respiratory conditions, and several abnormal laboratory markers were associated with the development of thrombosis. While thrombotic events occurred in 13% of the patients with a severe respiratory condition, those events still occurred in 2.5% of the patients who did not require oxygen therapy. Elevated D-dimer and ferritin levels on admission were independent risk factors of thrombosis (adjusted odds ratio 9.39 and 3.11, 95% confidence interval 2.08–42.3, and 1.06–9.17, respectively). Of the thrombotic events, 22 were venous, whereas 20 were arterial. While patients with thrombosis received anticoagulation and antiinflammatory therapies with a higher proportion, the mortality rate, organ dysfunctions, and bleeding complications in these patients were higher than those without thrombosis. The incidence of thrombosis in COVID-19 became less frequent over time, such as during the replacement of the earlier strains of SARS-CoV-2 by VOC/VOI and during increased use of anticoagulatory therapeutics.

**Conclusion:** This study elucidated that elevated D-dimer and ferritin levels are useful biomarkers of thrombosis in COVID-19 patients. The comparable incidence of arterial thrombosis with venous thrombosis and the development of thrombosis in less severe patients required further considerations for the management of Japanese patients with COVID-19. Further studies would be required to identify high-risk populations and establish appropriate interventions for thrombotic complications in COVID-19.

## Introduction

Coronavirus disease 2019 (COVID-19), caused by severe acute respiratory syndrome coronavirus 2 (SARS-CoV-2), has quickly spread worldwide and has yet to be brought under control. Although the major cause of death is respiratory failure, a wide range of unique complications has been observed in COVID-19 patients, including macrophage activating syndrome, multisystem inflammatory syndrome in children (MIS-C), and thrombosis (1, 2). Various studies have revealed incidences of deep vein thromboses and pulmonary embolisms in patients with severe disease, especially in patients admitted to the intensive care unit (ICU) (3–5).

Coagulopathy is a particular pathology observed with COVID-19 (6, 7). Various types of innate immune activation, including cytokine release, endothelial cell activation, and enhanced neutrophil extracellular traps, have been seen simultaneously in patients with thrombotic complications, indicating that the immunothrombosis might play a key role in the development of thrombosis in COVID-19 (1, 8–10). D-dimer is known to be markedly elevated over the clinical course of COVID-19 and is a risk factor for poor prognosis (2, 11, 12). Microembolisms in the pulmonary capillaries could impair oxygenation and induce so-called “silent hypoxia,” which was seen in the early stages of COVID-19 as a discrepancy of oxygen and carbon dioxide levels (12).

The worldwide pandemic is still ongoing and has even expanded in some countries with emerging SARS-CoV-2 variants of concern (VOC) and variant of interest (VOI), such as alpha variant (B.1.1.7), beta variant (B.1.351), gamma variant (P.1), delta variant (B.1.617.2), and R.1 variant (13). Since these variants carry various mutants, the properties of virus would be changed in terms of transmissibility, disease severity, the performance of vaccines, and complications (14). The clinical characteristics of COVID-19 caused by VOC/VOI have not yet been fully elucidated, including the incidence of thrombosis.

So far, there is limited evidence about the incidence of thrombosis and the association of biomarkers in Japanese population, except a few (15–18). The objective of the current study was to identify the risk factors for the development of thrombosis. In this retrospective study, we surveyed the clinical characteristics of thromboses in patients hospitalized for COVID-19 from the early phase of the pandemic to the recent cases caused by VOC/VOI in Japan. Here, we report the incidence of venous and arterial thrombosis, the pattern of sequential changes in biomarkers before the development of thrombosis, the incidence of thrombosis, and the proportion of VOC/VOI in a relatively large number of the patients at a single center.

## Materials and Methods

### Study Design/Setting

This single-center retrospective observational study included all patients with confirmed COVID-19 who were admitted at Tokyo Medical and Dental University (TMDU) hospital, a Japanese tertiary emergency hospital in an urban setting. The diagnosis of COVID-19 was made by detecting N gene of SARS-CoV-2 described previously (19). Briefly, clinical samples were obtained from oropharyngeal swabs and were analyzed with real-time reverse-transcription PCR (RT-PCR) using the method established in the National Institute of infectious disease in Japan (20), or 2019 Novel Coronavirus Detection Kit (Shimadzu Corporation, Kyoto, Japan). As the proportion of SARS-CoV-2 variants carrying either N501Y, E484K, and L452R mutations was expanded, RT-PCR was performed using VirSNiP SARS-CoV-2 (TIB Molbiol, Berlin, Germany). The mutation types were determined by the melting temperature.

This study protocol was approved by the ethics committees of TMDU. Reference ID was M2020-027.

### Data Collection and Definition

All the data, namely viral strains, demographics, disease severity, comorbidities, laboratory data, treatment, and outcomes were collected from electronic medical records.

Disease severity was measured on admission and at times of the most severe clinical condition. Based on the “guideline of the medical treatment of the noble coronavirus” presented by Japanese ministry of Health, Labor, and Welfare (21), disease severity was defined as follows: mild, for patients who do not need oxygen therapy; moderate, for patients who need oxygen of equal or <5 L/min; and severe, for patients who need oxygen of more than 5 L/min or mechanical ventilation.

Anticoagulation therapy was considered in the patients with severe disease, elevated D-dimer (>5 μg/mL), and/or in those who received baricitinib. Unfractionated heparin at therapeutic doses was defined as the dose determined in reference to the activated partial thromboplastin time (APTT). Unfractionated heparin at prophylactic doses was defined as a fixed dose (≤10,000 U/day), regardless of the APTT.

Acute myocardial infarctions (AMI), ST-elevation myocardial infarction and non-ST-elevation myocardial infarction, were diagnosed based on dynamic changes in cardiac enzyme levels, electrocardiogram findings, and ultrasound cardiography findings. Cerebral infarction was diagnosed based on symptoms and magnetic resonance imaging findings. Other arterial thromboses were diagnosed by contrast-enhanced CT scanning. Venous thromboembolism was diagnosed by contrast-enhanced computed tomography scanning, ultrasonography, autopsy, or clinically evident cases (i.e., the coincidence of rapid deterioration of the respiratory condition and marked elevation of D-dimer levels). Major bleeding was defined according to the International Society on Thrombosis and Haemostasis as overt bleeding which includes the following: fatal bleeding, symptomatic bleeding in a critical area or organ (i.e., intracranial, intraspinal, intraocular, retroperitoneal, intraarticular, pericardial, intramuscular with compartment syndrome), bleeding causing a fall in hemoglobin level of 20 g/l or more, or leading to transfusion of two or more units of whole blood or red cells (22).

### Statistical Analysis

We compared the distributions of continuous and categorical variables between the two groups using the Student's *t*-test (or Mann–Whitney *U*-test) and Chi-squared test (or Fisher's exact test), respectively. Univariate and multivariate logistic regression analyses were used to examine the impacts of the following variables on the development of arterial thrombosis: older age (**≥**65 vs. <65 years old), severity on admission (severe vs. mild/moderate), D-dimer (**≥**0.71 vs. <0.71 μg/mL), CRP (**≥**3.95 mg/dL vs. <3.95 mg/dL), and ferritin (**≥**434 vs. <434 ng/mL). Variables included in the multivariate logistic model were selected with the known risk factors (older age, disease severity, and D-dimer) (23–25), and the biomarker of inflammation (CRP) and macrophage activating syndrome (ferritin) were selected as the variables of interest for this study. The median values in 516 patients we analyzed were chosen as cutoff points for the D-dimer, CRP, and ferritin. The odds ratio (OR) including 95% confidence interval (CI) were estimated. Statistical significance was defined as *p* < 0.05. All statistical analyses were performed using IBM SPSS, version 27 (IBM, Armonk, NY, USA).

## Results

### Clinical Characteristics of Patients With and Without Thrombosis

We analyzed 516 consecutive patients from April 1, 2020, to July 31, 2020. The mean age of the patients was 57.2 ± 17.5 years old, 358 patients (69%) were men, and the mean body mass index (BMI) was 24.3 ± 4.9 kg/m^2^. Disease severity on admission was mild in 279 (54%), moderate in 128 (25%), and severe in 109 (21%) patients (Supplementary Table 1).

Of those, 32 patients (5.3%) experienced 42 thromboembolic events. Multiorgan infarctions were identified simultaneously in five patients. The mean age was significantly older in patients with thrombosis (66.9 vs. 56.1 years). In terms of the respiratory condition on admission, patients with thrombosis comprised a higher proportion of severe category than those without (44 vs. 20%). While thrombotic events occurred in 13% of the patients with a severe respiratory condition, these events still occurred in 2.5% of the patients who did not require oxygen supply (Table 1).

**Table 1:** Clinical characteristics of the patients complicated with or without thrombosis in the cohort.

	**All patients**	**Thrombosis**	**Non thrombosis**	*p-*value^*^
	**(*N* = 516)**	**(*N* = 32)**	**(*N* = 484)**	
**Baseline characteristics**				
Age, mean (SD)	57.2 (17.5)	66.9 (12%)	56.6 (17.6)	**<0.001**
≥65 years, *n* (%)	198 (38%)	22 (69%)	176 (36%)	**<0.001**
Male gender, *n* (%)	358 (69%)	25 (78%)	333 (69%)	0.33
Body mass index (kg/m^2^), mean (SD)	24.3 (4.9)	24.3 (4.0)	24.3 (5.0)	0.99
≥30 kg/m^2^, *n* (%)	53 (10%)	2 (6.3%)	51 (11%)	0.56
Current smoker, *n* (%)	87	2 (6.3%)	85 (18%)	0.13
Severity on admission, *n* (%)				**<0.001**
Mild	279 (54%)	7 (21.9%)	272 (56%)	
Moderate	128 (25%)	11 (34%)	117 (24%)	
Severe	109 (21%)	14 (44%)	95 (20%)	
**Comorbidities**				
Diabetes mellitus, *n* (%)	112 (22%)	5 (16%)	107 (22%)	0.39
Hypertension, *n* (%)	173 (34%)	9 (28%)	164 (34%)	0.5
Hyperlipidemia, *n* (%)	82 (16%)	3 (9.4%)	79 (16%)	0.3
COPD, *n* (%)	14 (2.7%)	1 (3.1%)	13 (2.7%)	0.60
Asthma, *n* (%)	37 (8.1%)	0 (0%)	37 (8.1%)	0.16
eGFR <60 ml/min/1.73m^2^, *n* (%)	149 (29%)	16 (50%)	133 (28%)	**0.006**
History of coronary artery disease, *n* (%)	19 (3.7%)	2 (6.3%)	17 (3.5%)	0.33
History of cerebral infarction, *n* (%)	24 (4.7%)	4 (13%)	20 (4.1%)	0.05
History of malignancy, *n* (%)	70 (14%)	6 (19%)	64 (13%)	0.42
Active malignancy, *n* (%)	14 (2.7%)	1 (3.1%)	13 (2.7%)	0.60
Autoimmune disease, *n* (%)	30 (5.8%)	4 (13%)	26 (5.4%)	0.11
**Laboratory data on admission**				
White blood cell count (× 10^3^/μL), median (IQR)	5.4 (4.1–7.3)	9.0 (4.1–7.2)	5.4 (5.4–9.9)	**0.003**
Lymphocyte count (/μL), median (IQR)	968.1 (651–1,315)	867 (686–1,326)	987 (443–779)	**<0.001**
Hemoglobin (g/dL), median (IQR)	14.3 (12.9–15.4)	13.1 (12.9–15.5)	14.3 (11.6–14.7)	**0.01**
Platelet count (× 10^4^/μL), median (IQR)	18.8 (15.6–24)	20.7 (15.7–24.0)	19.2 (13.3–25.6)	0.42
CRP (mg/dL), median (IQR)	3.95 (0.88–9.6)	12.6 (0.78–8.72)	3.59 (7.58–16.0)	**<0.001**
LDH (U/L), median (IQR)	292 (213–393)	494 (212–388)	284 (320–474)	**<0.001**
Ferritin (ng/mL), median (IQR)	434 (208–918)	1,124 (193–848)	403 (508–1335)	**<0.001**
Creatinine (mg/dL), median (IQR)	0.84 (0.66–1.03)	1.91 (0.65–1.00)	0.83 (0.69–1.28)	0.11
PT (second), median (IQR)	11.1 (10.5–12.0)	12.9 (10.4–11.9)	11.1 (11.0–14.1)	**<0.001**
APTT (second), median (IQR)	32.0 (29.3–35.3)	35.5 (29.3–35.1)	31.9 (29.1–38.5)	0.18
D-dimer (μg/mL), median (IQR)	0.71 (0.50–1.60)	11.6 (0.5–1.5)	0.67 (1.2–11.3)	**<0.001**
Fibrinogen (mg/dL), median (IQR)	480 (378–565)	570 (376–558)	471 (465–699)	**<0.001**
FDP (μg/mL), median (IQR)	3.35 (2.5–5.7)	36.0 (2.5–5.3)	3.2 (3.5–20.0)	**<0.001**

* *Chi-squared test or Fisher's extract test for categorical variables, student's t-test for age and Body mass index and Mann-Whitney U test for laboratory data. Statistically significant p < 0.05 values are in bold. SD, standard deviation; IQR, Interquartile range; COPD, Chronic Obstructive Pulmonary Disease; CRP, C reactive protein; LDH, lactate dehydrogenase; PT, prothrombin time; APTT, activated partial thromboplastin time; FDP, fibrinogen degradation products*.

Among the underlying comorbidities, impaired renal function (eGFR <60 ml/min/1.73 m^2^) was more frequently observed in thrombotic cases (50 vs. 28%). Patients experiencing thrombotic events had significantly higher white blood cell counts and higher levels of C-reactive protein (CRP), lactate dehydrogenase (LDH), ferritin, D-dimer, fibrinogen, and fibrinogen degradation products (FDP), prolonged prothrombin time (PT), and lower lymphocyte counts and hemoglobin levels than those without thrombotic events on admission (Table 1).

Multivariate logistic regression models revealed that elevated D-dimer levels over 0.71 μg/mL (adjusted odds ratio 9.39, 95% confidence interval 2.08–42.3) and ferritin levels over 434 ng/mL (adjusted odds ratio 3.11, 95% confidence interval 1.06–9.17) were independent predictive factors for the thrombosis (Table 2).

**Table 2:** Univariate analysis and multivariate logistic regression analysis adjusted for the variables.

**Variables**	**Univariate analysis**			**Multivariate analysis**		
	**OR**	**95% CI**	***p*-value**	**OR**	**95% CI**	***p*-value**
Older age	3.82	1.62–8.99	0.002	1.64	0.70–3.82	0.25
Severity	3.36	1.54–7.34	**<0.001**	1.23	0.52–2.87	0.64
D-dimer	10.7	3.22–35.7	**<0.001**	9.39	2.08–42.3	**0.004**
CRP	4.71	1.90–11.6	**<0.001**	1.91	0.64–5.69	0.25
Ferritin	5.23	1.96–14.0	**<0.001**	3.11	1.06–9.17	**0.04**

*Statistically significant p < 0.05 values are in bold. CI, confidence interval*.

### Arterial vs. Venous Thrombosis

**Figure 1:**
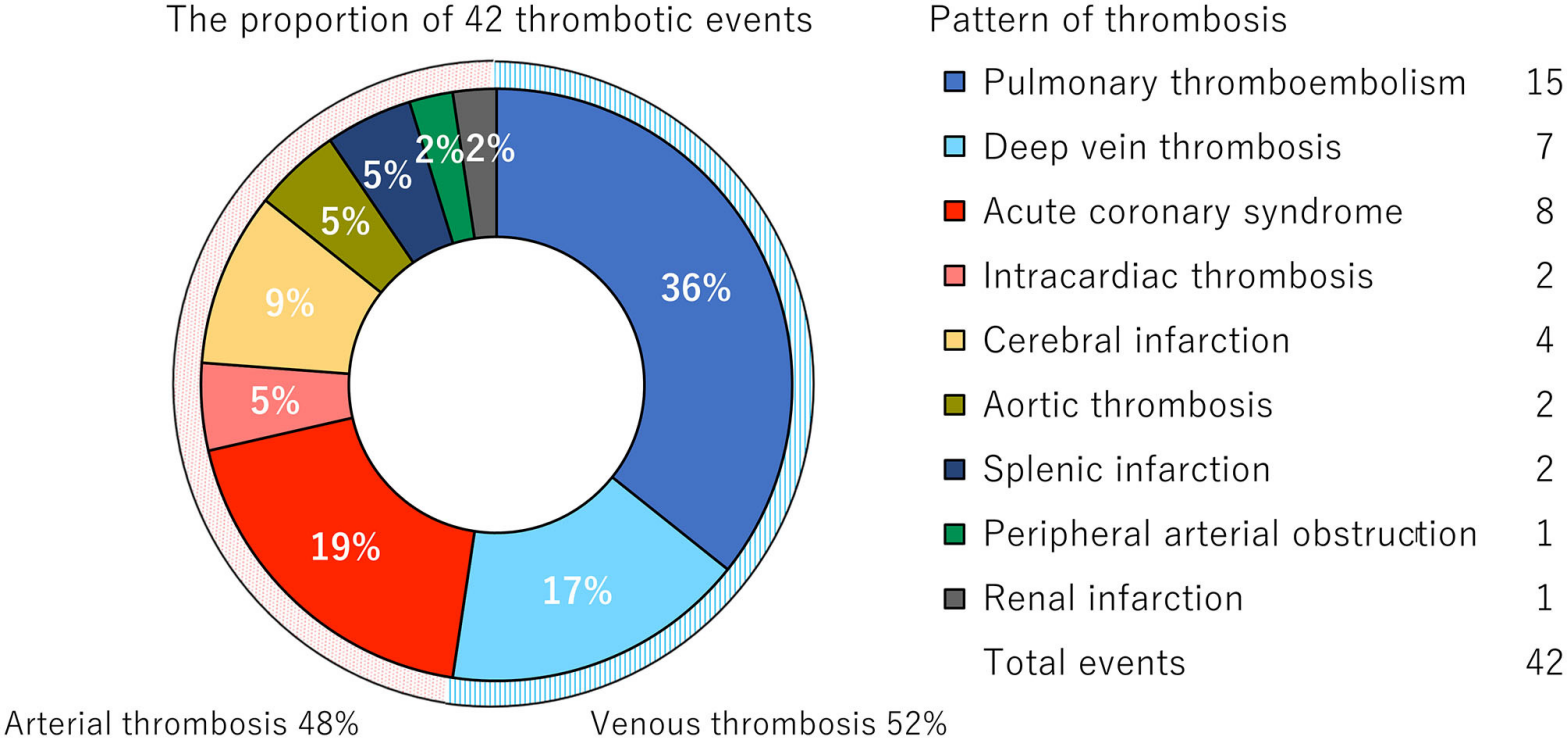
Distribution of the thrombotic events. Pie chart represents the proportion of 42 thrombotic events complicated in 32 patients.

As reported previously in COVID-19 associated thrombosis (26, 27), the unique patterns of thromboses were observed in our study (Figure 1, Supplementary Table 2). Of the 42 thrombotic events, 20 (48%) events were arterial thrombosis. Among the arterial thrombotic events, acute coronary syndrome was the most frequently observed in eight and cerebral infarction was observed in four. A total of five atypical arterial thrombotic cases were diagnosed: two cases of cooccurrence of myocardial infarction and cardiac ventricular thrombosis; a cooccurrence of splenic and renal infarction; and of note, floating thrombus in the ascending aorta was detected in two cases, resulting in the infarction of brain and spleen, or left extremity, respectively (Supplementary Figure 1). Out of 22 (52%) venous thrombotic events, 11 were complicated with pulmonary thrombosis without deep vein thrombosis (DVT), while four were identified as the cooccurrence of DVT and pulmonary embolism. The other three were complicated DVT alone. No cooccurrence of arterial and venous thrombosis was observed.

There was no difference in the clinical characteristics between patients with arterial or venous thrombotic events, except for the peak D-dimer levels (Table 3). The median durations from the disease onset to the development of an arterial or a venous thrombotic event were 13 and 16 days, respectively. The clinical significances were variable depending on the disease conditions (Supplementary Table 2). The deterioration of respiratory condition was observed in nine cases after the development of thrombosis. Among the 32 cases, 21 received escalation of anticoagulation strategy, whereas the others did not, based on their clinical conditions such as bleeding complications or poor prognosis.

**Table 3:** Clinical course of the patients complicated with arterial thrombosis or venous thromboembolism.

	**Arterial thrombosis (*N* = 14)**	**Venous thromboembolism (*N* = 18)**	***p-*value**
Baseline characteristics			
Age, mean (SD)	70 (10.6)	64.4 (12.6)	0.18
Male gender, *n* (%)	12 (86%)	13 (72%)	0.43
Body mass index (kg/m^2^), mean (SD)	26.4 (3.1)	22.9 (3.9)	0.15
ICU admission, *n* (%)	5 (36%)	8 (62%)	0.62
Severity on admission, *n* (%)			0.68
Mild	4 (29%)	3 (17%)	
Moderate	4 (29%)	6 (33%)	
Severe	6 (43%)	9 (50%)	
Days from onset to thrombosis, median (IQR)	13 (6–20)	16 (14–18)	0.15
Comorbidities			
Diabetes mellitus, *n* (%)	2 (14%)	3 (16.7%)	1.00
Hypertension, *n* (%)	4 (29%)	5 (28%)	1.00
Hyperlipidemia, *n* (%)	1 (7.1%)	2 (11%)	1.00
COPD, *n* (%)	1 (7.1%)	0 (0%)	0.44
Asthma, *n* (%)	0 (0%)	0 (0%)	0.44
eGFR <60 ml/min/1.73 m^2^, *n* (%)	9 (64%)	7 (39%)	0.15
History of coronary artery disease, *n* (%)	2 (15%)	0 (0%)	0.18
History of cerebral infarction, *n* (%)	1 (5.6%)	3 (21%)	0.30
History of malignancy, *n* (%)	2 (14%)	4 (22%)	0.67
Active malignancy, *n* (%)	0 (0%)	1 (5.6%)	1.00
Autoimmune disease, *n* (%)	2 (11%)	2 (11%)	1.00
Laboratory data at the onset of thrombosis			
White blood cell count (× 10^3^/μL), median (IQR)	7.4 (5.5–10.9)	8.6 (6.7–11.3)	0.46
Hemoglobin (g/dL), median (IQR)	12.3 (10.9–14.1)	12.7 (10.0–13.9)	0.78
Platelet count (× 10^4^/μL), median (IQR)	24.5 (9.0–31.1)	26.9 (13.0–33.6)	0.61
CRP (mg/dL), median (IQR)	1.67 (0.52–6.16)	7.20 (1.13–16.1)	0.10
Ferritin (ng/mL), median (IQR)	392 (229–932)	1,054 (592–1,610)	0.06
D-dimer (μg/mL), median (IQR)	5.0 (3.26–13.2)	16.8 (9.2–41.3)	**0.01**
Fibrinogen (mg/dL), median (IQR)	303 (155–510)	378 (253–585)	0.47
FDP (μg/mL), median (IQR)	8.8 (6.6–14.8)	24.7 (7.1–42.6)	0.15
PT (second), median (IQR)	11.8 (10.6–13.3)	13.2 (11.7–16.0)	0.12
APTT (second), median (IQR)	35.0 (27.1–39.4)	38.6 (28.5–48.4)	0.21
Outcomes			
Death, *n* (%)	2 (14%)	6 (33%)	0.41

*Statistically significant p < 0.05 values are in bold. SD, Standard Deviation; IQR, Interquartile range; COPD, Chronic Obstructive Pulmonary Disease; CRP, C reactive protein; LDH, lactate dehydrogenase; PT, prothrombin time; APTT, activated partial thromboplastin time; FDP, fibrinogen degradation products*.

### Treatments and Outcomes

Clinical courses of the patients are summarized in Table 4. Regarding the treatments in those with or without thrombosis, glucocorticoid (75 vs. 52%), tocilizumab (28 vs. 6.0%), and unfractionated heparin at therapeutic doses (47 vs. 7.9%) were used more frequently in patients complicated with thrombosis. Thirteen out of the 32 patients with thrombosis developed thrombosis even under prophylactic or therapeutic anticoagulation therapy, indicating that thrombotic events could not be prevented by anticoagulation in some patients. Among the 13 cases, seven were arterial thrombosis and six were venous.

**Table 4:** Clinical course of the first cohort patients complicated with or without thrombosis.

	**All patients**	**Thrombosis**	**Non-Thrombosis**	***p-value***
	**(*N* = 516)**	**(*N* = 32)**	**(*N* = 484)**	
**Non-Pharmacological intervention**				
ICU admission, *n* (%)	109 (21%)	13 (41%)	96 (20%)	**0.005**
Oxygen therapy, *n* (%)	316 (61%)	29 (91%)	289 (60%)	**<0.001**
Mechanical ventilation, *n* (%)	136 (26%)	19 (60%)	117 (24%)	**<0.001**
Renal replacement therapy, *n* (%)	33 (6.4%)	5 (16%)	28 (5.8&)	**0.045**
**Maximum severity**, ***n*** **(%)**				**<0.001**
Mild	186 (36%)	2 (6.3%)	184 (38%)	
Moderate	183 (36%)	8 (25%)	175 (36%)	
Severe	147 (28%)	22 (69%)	125 (26%)	
**Laboratory data at the peak severity**				
CRP (mg/dL), median (IQR)	6.22 (2.08–13.9)	14.6 (10.3–21.8)	5.84 (1.95–12.6)	**<0.001**
D-dimer (μg/ml), median (IQR)	2.01 (0.76–6.1)	16.9 (7.21–48.7)	1.80 (0.70–4.42)	**<0.001**
**Treatment**				
Glucocorticoid therapy, *n* (%)	274 (53%)	24 (75%)	250 (52%)	**0.010**
Tocilizumab, *n* (%)	38 (7.4%)	9 (28%)	29 (6%)	**<0.001**
DOAC, *n* (%)	26 (5.0%)	10 (31%)	16 (3.3%)	**<0.001**
Prophylactic anticoagulation dose, *n* (%)	131 (25%)	11 (34%)	120 (25%)	0.23
Therapeutic anticoagulation dose, *n* (%)	53 (10%)	15 (47%)	38 (7.9%)	**<0.001**
**Outcomes**				
Major bleeding, *n* (%)	15 (2.9%)	7 (22%)	8 (1.7%)	**<0.001**
Death, *n* (%)	37 (7.2%)	8 (25%)	29 (6.0%)	**<0.001**
Length of hospitalization (days)	16.5 (8–20)	25.5 (16–36)	15.5 (8–19)	**<0.001**

*Statistically significant p < 0.05 values are in bold. IQR, interquartile range; ICU, intensive care unit; CRP, C-reactive protein; DOAC, direct oral anticoagulant*.

In patients with thrombosis, the percentages of patients who needed oxygen therapy (91 vs. 60%), mechanical ventilation (60 vs. 24%), the rate of mortality (25 vs. 6.0%), and bleeding complications (22 vs. 1.7%) were significantly higher than in those without.

### Clinical Course and Changes in Coagulation Marker Over Time

**Figure 2:**
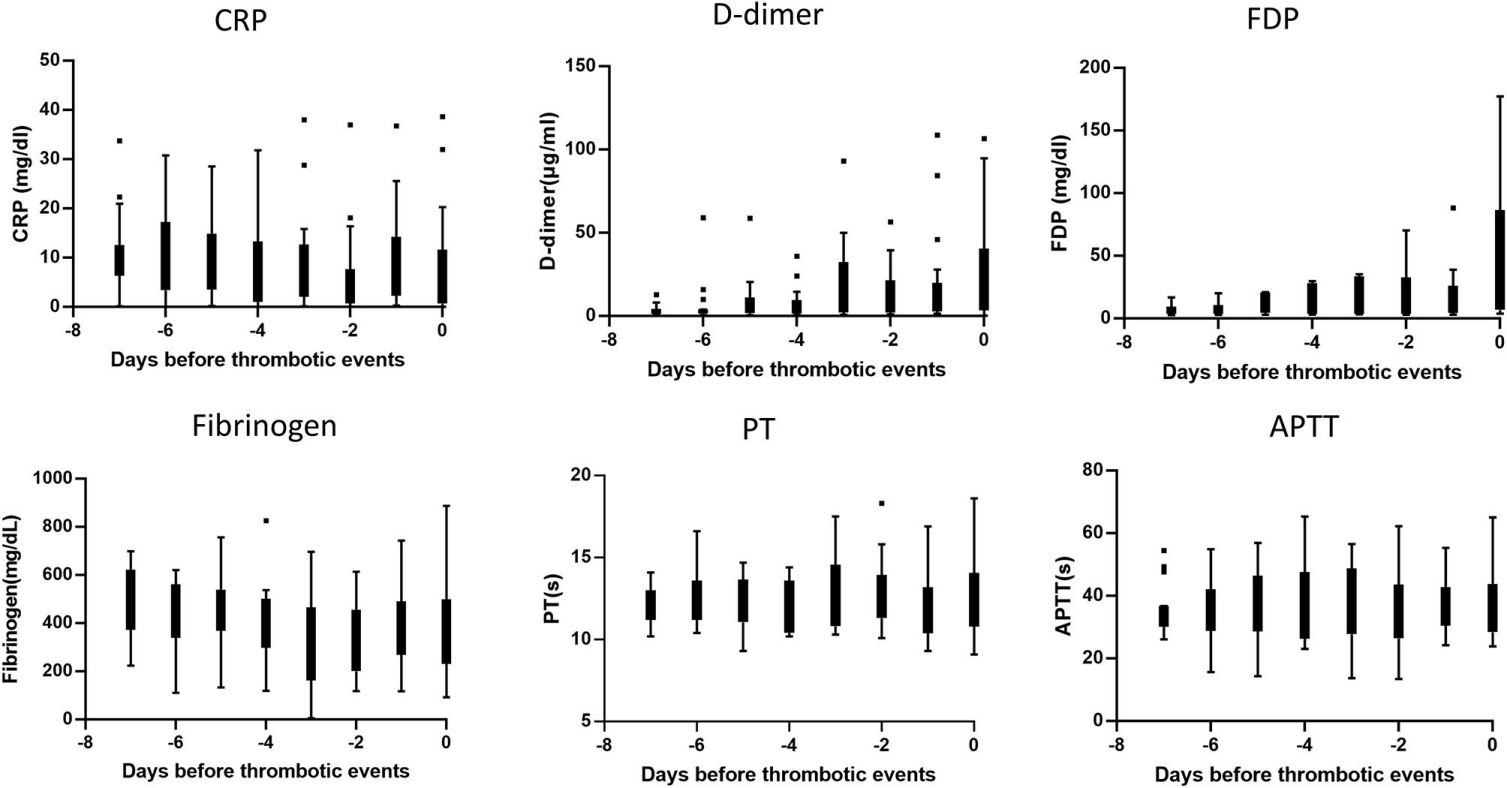
Sequential changes of the biomarkers before the development of thrombosis. Each graph indicated the sequential change of individual biomarkers until the day of thrombotic events. The box-whisker plot indicated median and upper/lower quartile of the data of 32 patients complicated with thrombosis.

To explore the underlying mechanism of thrombosis in COVID-19, we analyzed the sequential changes in the balance of coagulation and fibrinolysis biomarkers and inflammatory status in the 32 patients (Figure 2). In most of the thrombotic patients, FDP and D-dimer levels began to be elevated several days before the occurrence of thrombosis. When the respiratory condition was sequentially evaluated, we found that thrombosis occurred during exacerbation in seven cases, sustaining in 16 cases, and improvement in nine cases. Notably, in the last group of cases, the temporal discrepancy of inflammation and coagulopathy was apparent, namely, inflammation peaked 1 week before the onset of thrombosis, whereas FDP and D-dimer levels were elevated simultaneously with the onset of thromboses (Supplementary Figure 2).

### Thrombosis in COVID-19 Caused by SARS-CoV-2 VOC/VOI or Non-COV/VOI

**Figure 3:**
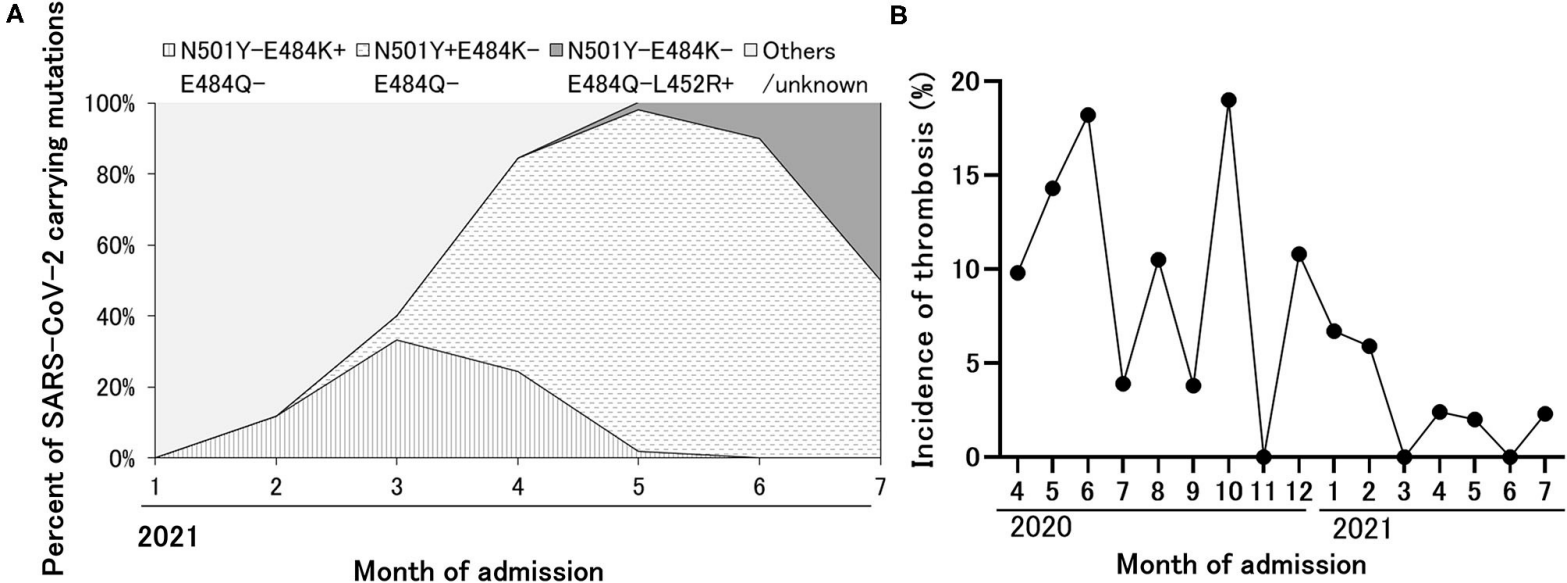
The replacement of SARS-CoV-2 strain to VOC/VOI during the observation period and decreased incidence of thrombosis over time. **(A)** The expansion of variant strain of SARS-CoV-2 over time detected in the patients admitted with COVID-19. Based on the results of the epidemiologic analysis in Tokyo, the variant carrying E484K alone, N501Y alone, and L452R alone were considered as R.1 lineage variant, alpha variant, and delta variant, respectively. **(B)** The sequential change of the incidence of thrombosis during the observation period.

As we have reported, the ratio of VOC/VOI carrying N501Y or E484K mutation rapidly increased in our hospital during the observation period, and the proportion of VOC/VOI reached 100% by the end of April in 2021 (Figure 3A). Unexpectedly, the incidence of thrombosis showed decreased trend from March in 2021 (Figure 3B). Therefore, we compared the patient's characteristics of COVID-19 caused by SARS-CoV-2 VOC/VOI to those by non-VOC/VOI. We found that the proportion of severe category was similar (20 vs. 22%), whereas the incidence of thrombosis was less frequent in the COVID-19 caused by VOC/VOI than the others (1.3 vs. 8.2%). In addition, the younger age and high frequency of anticoagulation therapy at therapeutic doses were also observed in the patients with COVID-19 caused by VOC/VOI (Table 5).

**Table 5:** Comparison of the clinical characteristics of COVID-19 caused by SARS-CoV-2 VOC/VOI or non-VOC/VOI.

	**SARS-CoV-2 VOC/VOI**	**SARS-COV-2 non-VOC/VOI**	***p-value***
	**(*N* = 152)**	**(*N* = 364)**	
**SARS-CoV-2 VOC/VOI, n (%)**	152	–	
Carrying 501Y, 484E (alpha strain, VOC)	109 (72%)	–	
Carrying 501N, 484K (R.1 strain, VOI)	19 (13%)	–	
Carrying 501N, 484E, 452R (delta strain, VOC)	24 (16%)	–	
**Baseline characteristics**			
Age, mean (SD)	53 (15.9)	59.5 (17.4)	**0.001**
≥65 years, *n* (%)	38 (25%)	160 (44%)	**<0.001**
Male gender, *n* (%)	110 (72%)	248 (68%)	0.34
Body mass index (kg/m^2^), mean (SD)	24 (4.7)	24.5 (5.0)	0.32
≥30 kg/m^2^, *n* (%)	16 (11%)	37 (11%)	0.88
Current smoker, *n* (%)	31 (21%)	56 (18%)	*0*.45
**Severity on admission**, ***n*** **(%)**			0.87
Mild	83 (55%)	196 (54%)	
Moderate	39 (26%)	89 (25%)	
Severe	30 (20%)	79 (22%)	
**Comorbidities**			
Diabetes mellitus, *n* (%)	33 (22%)	79 (22%)	*1.00*
Hypertension, *n* (%)	48 (32%)	125 (34%)	*0.58*
Hyperlipidemia, *n* (%)	28 (18%)	54 (15%)	*0.31*
COPD, *n* (%)	4 (2.6%)	10 (2.7%)	*1.00*
Asthma, *n* (%)	15 (11%)	22 (6.2%)	*0.055*
Chronic kidney disease, *n* (%)	38 (25%)	111 (31%)	*0.21*
History of coronary artery disease, *n* (%)	6 (4.0%)	13 (3.6%)	*0.83*
History of cerebral infarction, *n* (%)	6 (4.0%)	18 (4.9%)	*0.63*
History of malignancy, *n* (%)	14 (9.3%)	56 (15%)	*0.07*
Active malignancy, *n* (%)	5 (3.3%)	9 (2.5%)	*0.56*
Autoimmune disease, *n* (%)	7 (4.6%)	23 (6.3%)	*0.46*
**Laboratory data at admission**			
White blood cell count (× 10^3^/μL), median (IQR)	5.2 (3.9–7.3)	5.6 (4.1–7.3)	0.24
Lymphocyte count (/μL), median (IQR)	927 (630–1,188)	998 (672–1,387)	**0.02**
Hemoglobin (g/dL), median (IQR)	14.3 (13.1–15.6)	14.2 (12.7–15.4)	0.17
Platelet count (× 10^4^/μL), median (IQR)	17.6 (14.9–21.7)	19.9 (16.3–25.3)	**<0.001**
CRP (mg/dL), median (IQR)	3.41 (1.13–8.01)	4.23 (0.79–9.98)	0.35
LDH (U/L), median (IQR)	305 (229–424)	285 (212–391)	0.19
Ferritin (ng/mL), median (IQR)	493 (198–1,073)	427 (213–828)	0.28
Creatinine (mg/dL), median (IQR)	0.84 (0.66–1.01)	0.84 (0.65–1.03)	0.78
PT (second), median (IQR)	10.9 (10.1–11.6)	11.2 (10.5–12.1)	**<0.001**
APTT (second), median (IQR)	32.7 (29.8–36.3)	31.9 (29.1–35.0)	0.14
D-dimer (μg/mL), median (IQR)	0.59 (0.50–1.27)	0.78 (0.50–1.69)	0.83
Fibrinogen (mg/dL), median (IQR)	465 (389–546)	485 (373–578)	0.44
FDP (μg/mL), median (IQR)	2.5 (2.5–3.5)	4 (2.6–6.1)	**<0.001**
**Treatment**			
DOAC, *n* (%)	8 (5.3%)	18 (4.9%)	0.88
Prophylactic doses of anticoagulation therapy, *n* (%)	43 (28%)	88 (24%)	0.33
Therapeutic doses of anticoagulation therapy, *n* (%)	29 (19%)	24 (6.6%)	**<0.001**
**Outcome**, ***n*** **(%)**			
Thrombosis, *n* (%)	2 (1.3%)	30 (8.2%)	**0.003**
Major bleeding, *n* (%)	4 (2.6%)	11 (3.0%)	1.00
Death, *n* (%)	9 (5.9%)	28 (7.7%)	0.48

*Statistically significant p < 0.05 values are in bold. VOC, variant of concern; VOI, variant of interest; COPD, chronic obstructive pulmonary disease; CRP, C-reactive protein; LDH, lactate dehydrogenase; PT, prothrombin time; APTT, activated partial thromboplastin time; FDP, fibrinogen degradation products; DOAC, direct oral anticoagulant*.

## Discussion

Our study revealed that thrombotic complications developed more frequently in patients with a severe respiratory disease on admission and were associated with death, consistent with the previous findings in the Japanese population (16). Additionally, elevated D-dimer and ferritin levels on admission were the risk factors for the development of thrombosis. Arterial and venous thrombotic complications occurred at similar rates. Of note, thrombotic complications developed even in cases with mild disease severity and not always at the peak of the course of the disease. The incidence of thrombosis decreased over time and might be less frequent in COVID-19 caused by VOC/VOI than the earlier strains, while the concomitant treatments including anticoagulation were different according to the in-hospitalized period.

We have identified that elevated D-dimer and ferritin levels on admission are independent risk factors for the development of thrombosis in COVID-19 patients by multivariate analysis. Several reports have revealed that disease severity and the elevation of D-dimer levels is a predictive factor of thrombosis (23–25). While ferritin was assumingly one of the predictive factors of developing acute respiratory distress syndrome of COVID-19 (28–30), we demonstrated for the first time that the elevation of ferritin was also an independent risk factor of thrombosis in the Japanese population with COVID-19. Since ferritin is induced with various inflammatory mediators including TNF, IL-1, IL-6, and IL-18, the elevation of ferritin level is a surrogate marker in macrophage activation in various diseases (31, 32). Various inflammatory mediators are released during the disease progression in COVID-19, where the innate immune activation is presumably the underlying mechanism of thrombosis, namely immunothrombosis (10, 33). Accordingly, we recommend evaluating ferritin levels as a biomarker of immunothrombosis for the management of patients with COVID-19.

Our study showed the comparable incidence of arterial and venous thrombotic events associated with COVID-19 in Japan, while a previous meta-analysis or systematic review revealed a high frequency of venous thrombosis (23%) (4) with relatively low frequencies of arterial thrombosis (4.4%) (27) in critically ill patients with COVID-19 in Western countries. Our findings were consistent with previous multicenter questionnaire surveys in Japan (17) and a recent report from Singapore (34). Accordingly, relatively higher incidences of arterial thrombosis might be a unique feature in the Asian population, but ethnic differences were minimum with respect to venous thrombosis (35). Consistent with the previous reports, thrombotic complications were developed more frequently in patients with severe respiratory disease than the others. These findings would be associated with more severe clinical conditions of patients with thrombosis than those without thrombosis. Although the causal relation was unproven, the association between the epidemic of COVID-19 infection and the increase of out-of-hospital death due to cardiac disease or pulmonary thrombosis were reported, suggesting the possibility that venous or arterial thrombosis is one of the causes of sudden death observed in COVID-19 (36–38). Given the favorable outcomes that prophylactic anticoagulation reduced mortality in the initial observational study (39), guidelines from multiple organizations recommended consistently that hospitalized patients should receive prophylactic dose of anticoagulation therapy (40–42). Nevertheless, the incidence of thrombotic complications was still high in the critically ill patients with COVID-19, even though all patients received standard-dose thromboprophylaxis (5). Additionally, our results suggested that optimal prophylactic treatment needs to be established for the prevention of arterial thrombosis. So far, no preventive effects of aspirin against arterial thrombosis have been demonstrated according to some meta-analyses (43, 44). While the optimization of anticoagulation therapy has been eagerly examined (45–47), concomitant treatment targeting immune activation would be beneficial (48, 49) not only for the pulmonary disease of the patients but also for the prevention of thrombosis (33). Further investigations to identify high-risk patients and to develop effective prophylaxis would be required to prevent thrombosis caused by COVID-19.

Coagulation abnormality is one of the predictive factors for poor prognosis in COVID-19. Many reports indicated the dysregulation of coagulation systems in fatal cases, and coagulation abnormalities exacerbated the progression of the disease. The elevation of D-dimer on admission was reported in several studies, suggesting that an abnormal activation of the coagulation system had already occurred in the early phase of COVID-19. Several recent reports have suggested pathological functions of vascular endothelial dysfunction in the thrombosis of COVID-19 (49, 50). The complication of floating thrombi in the ascending aorta and pulmonary thromboembolism without DVT are known as characteristic patterns of thrombosis observed in COVID-19 (27). Similar findings were also observed in our study. After the infection of type II pneumocytes by SARS-CoV-2, cytokine production and neutrophil extracellular traps from migrated neutrophils might induce the activation of vascular endothelial cells, which results in immunothrombosis. Although D-dimer levels remained within the normal range in patients post COVID-19, the levels of factor VIII and PAI-1 were elevated after 4 months (51), suggesting the continuous activation of the vascular endothelium. Although SARS-CoV-2 had been considered to infect vascular endothelial cells in the early report (52), such direct infection is now considered a rare condition (53–55).

At the beginning of the observation period, all infected patients were required to be hospitalized regardless of their disease severity in Japan. Therefore, our data included a relatively high percentage of less severe patients. Such circumstances enabled us to assess the natural course of the disease and resultant complications during the early phase of the COVID-19. Our study revealed that thrombosis could occur in patients who did not require oxygen therapy, which would contribute to updating the management of cases with mild disease severity, including outpatient checkups at non-medical institutions. According to our findings, we propose to measure D-dimer and ferritin levels on admission to identify high-risk patients and follow-up these parameters in such patients.

A decreased incidence of thrombosis was observed during a later period in our cohort. The results might reflect the differences in clinical characteristics among the SARS-CoV-2 strains and/or the increasing use of anticoagulation therapeutics. So far, no study has identified the incidence of thrombosis among patients infected with different SARS-CoV-2 strains. Recently, several reports described the promotion of thrombus formation *via* the activation of platelets (56), the induction of tissue factor (57), or PAI-1 (58) using virus particle of the Wuhan strain or recombinant S1 protein with D614G single mutation. Although the mutations carried by VOC/VOI might reduce its thrombogenic properties instead of the increased ACE2 binding affinity and the enhanced infectivity, it would be difficult to identify the reason for the decreased incidence of thrombosis from our observation. To clarify the property of the thrombogenic potential among different SARS-CoV-2 strain, further data accumulation is required.

We must admit several limitations in this study. Due to the retrospective nature of the study, the issue of residual confounding was unavoidable. The incidence of thrombosis may have been underestimated since not all cases underwent ultrasound or contrast CT as a screening. The history of venous thrombosis would be a risk factor for developing another thrombosis. However, few patients had a history of venous thrombosis in our study, indicating that we might not collect several medical histories appropriately. Since the therapeutic anticoagulation therapy might be beneficial in the high-risk patients of thrombosis, we could not associate the superiority of the therapeutic approach to the prophylactic approach because of the nature of the study. To minimize the selection bias, we analyzed all hospitalized patients consecutively. However, there are missing data about some biomarkers or follow-up data, and the data of patients transferred to another institute whose systemic condition was not fully recovered. Furthermore, our multivariate logistic regression model may be overloaded with respect to the number of independent variables due to the small sample size. These limitations restricted the generalizability of our observations.

In conclusion, we reported the detailed clinical characteristics of COVID-19 patients, including variants information, with a focus on the development of thrombosis. Further large-scale studies to identify high-risk populations and establish appropriate preventive and therapeutic interventions would be required in the management of thrombotic complications in COVID-19.

## Data Availability Statement

The original contributions presented in the study are included in the article/Supplementary Material, further inquiries can be directed to the corresponding author/s.

## Ethics Statement

This study protocol was approved by the Ethics Committees of Tokyo Medical and Dental University (TMDU). Reference ID was M2020-027. Written informed consent was not required for this study, in accordance with the local legislation and institutional requirements.

## Author Contributions

SY, TH, TM, TO, TT, and YMa designed the research. SO, TH, MA, DK, TM, TA, SS, TT, HT, YT, KT, STo, and AH analyzed the data. SO, TH, AE, and SY wrote the manuscript. YMi and SY supervised the manuscript. All authors contributed to the data collection.

## Funding

This work was supported by Japan Agency for Medical Research and Development (AMED) under grant number 21ek0410083h0002.

## Conflict of Interest

The authors declare that the research was conducted in the absence of any commercial or financial relationships that could be construed as a potential conflict of interest.

## Publisher's Note

All claims expressed in this article are solely those of the authors and do not necessarily represent those of their affiliated organizations, or those of the publisher, the editors and the reviewers. Any product that may be evaluated in this article, or claim that may be made by its manufacturer, is not guaranteed or endorsed by the publisher.

## Supplementary Material

Supplementary Figure 1 Floating thrombus in aortic arch. Single-phase contrast-enhanced CT shows a filling defect in the aortic arch (arrowhead).

Supplementary Figure 2 Sequential changes of the biomarkers before the development of thrombosis in the seven cases whose respiratory conditions were ameliorated. Each graph indicated the sequential change of individual biomarkers until the day of thrombotic events. The box-whisker plot indicated median and upper/lower quartile of the data of nine patients complicated with thrombosis whose respiratory conditions were ameliorated within a week.

Supplementary Table 1 Baseline characteristics of the patients in our cohort. SD, standard deviation; COPD, chronic obstructive pulmonary disease.

Supplementary Table 2 Detail pattern of thrombosis complicated in the individual cases with COVID-19. DVT, Deep vein thrombosis; PE, Pulmonary embolism; PCI, Percutaneous coronary intervention. Distal or proximal DVT was defined as whether thromboses were identified in the calf vein or more proximal deep veins (59). In terms of severity of PE, massive, sub-massive, or non-massive was defined as whether PE with sustained hypotension, PE with normotension but with either right ventricular dysfunction and myocardial necrosis, or PE with the absence of clinical markers of adverse prognosis that define massive or sub-massive PE (60).
