# Supplementary figures and images for: Arterial and Venous Thrombosis Complicated in COVID-19: A Retrospective Single Center Analysis in Japan
Source: Front Cardiovasc Med. 2021 Nov 19;8:767074. doi: 10.3389/fcvm.2021.767074 (PMC8639692; doi:10.3389/fcvm.2021.767074)

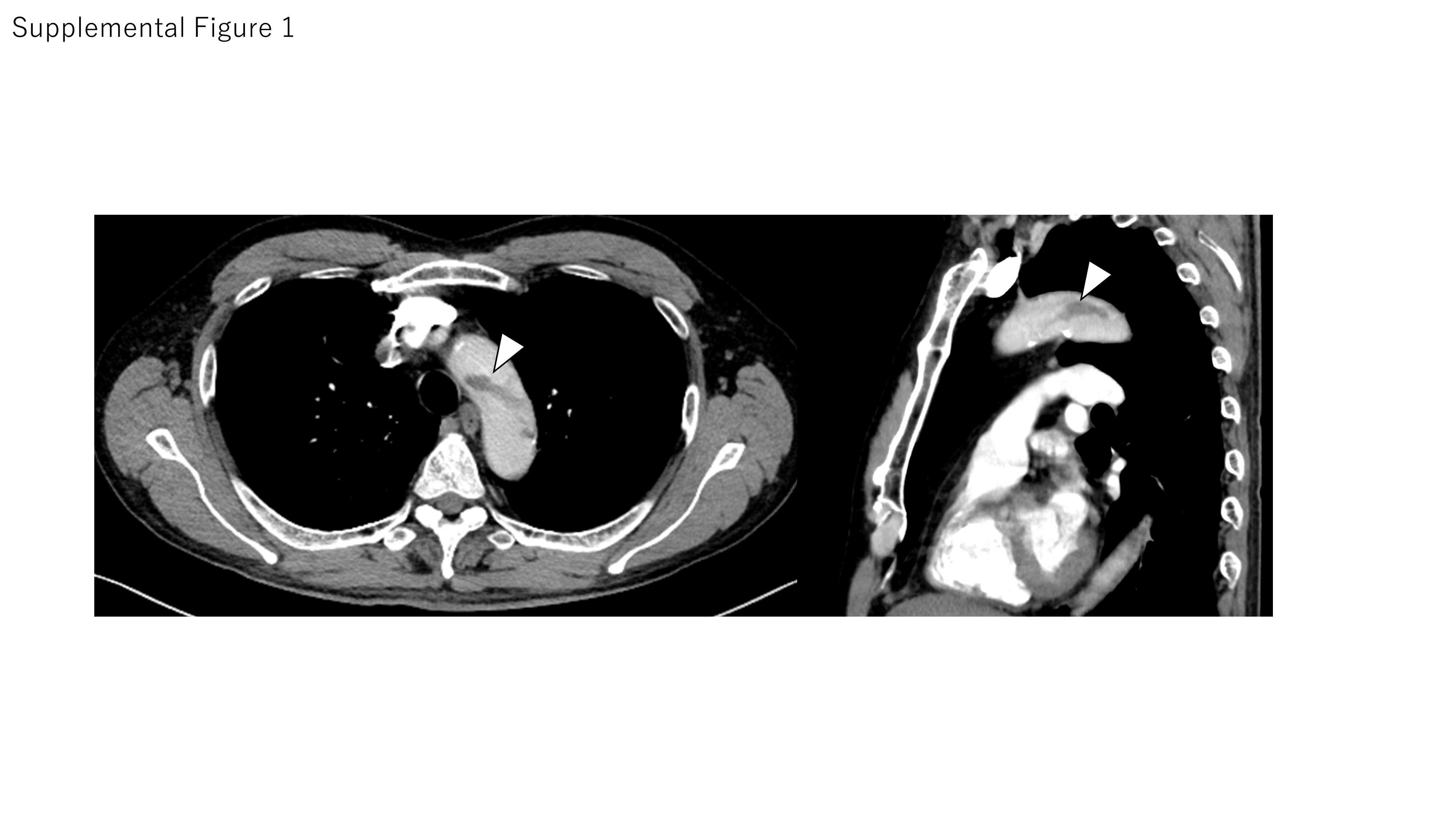

Supplement: Supplementary Figure 1 — Floating thrombus in aortic arch. Single-phase contrast-enhanced CT shows a filling defect in the aortic arch (arrowhead). [file Image_1.TIF]

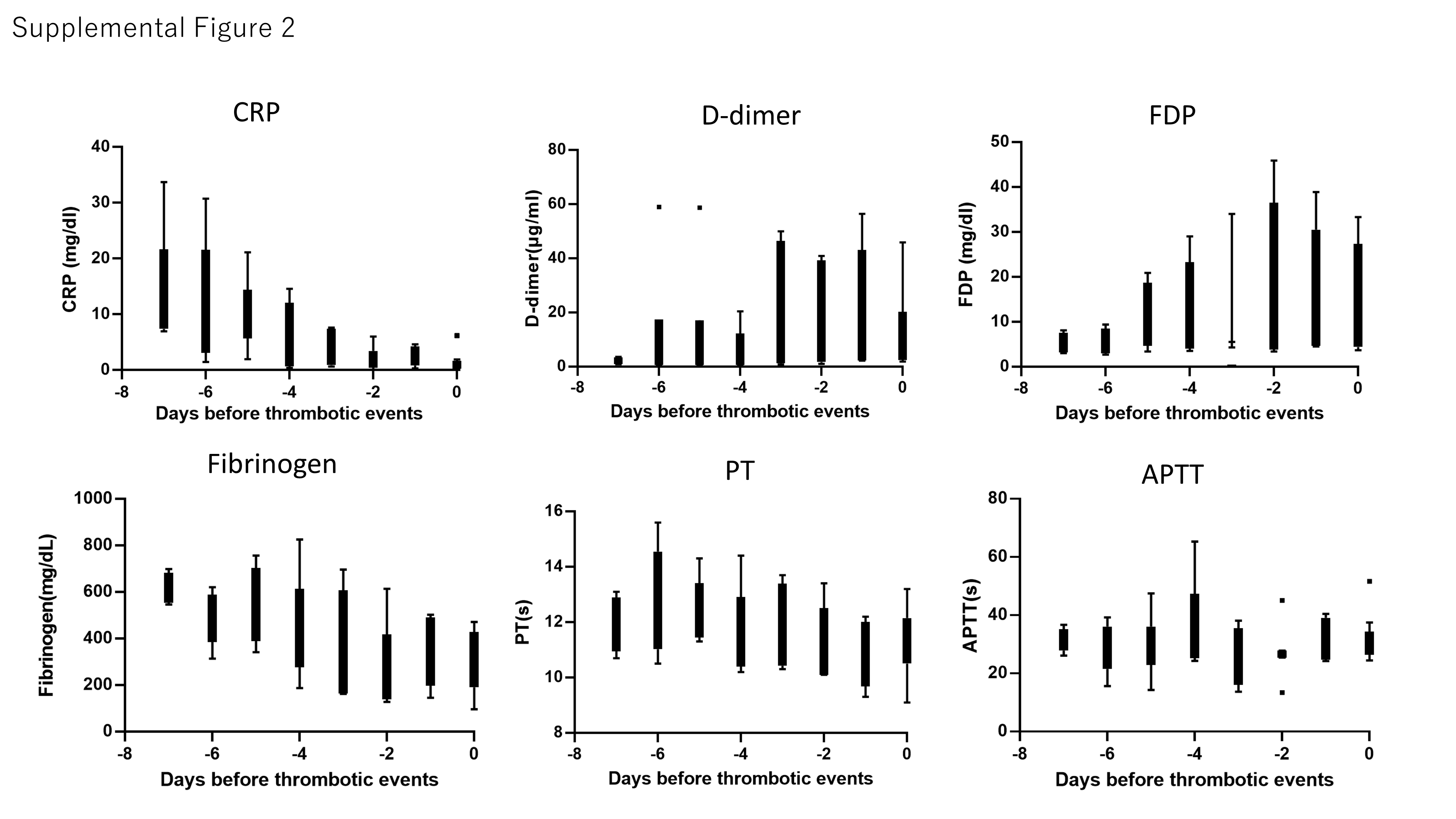

Supplement: Supplementary Figure 2 — Sequential changes of the biomarkers before the development of thrombosis in the seven cases whose respiratory conditions were ameliorated. Each graph indicated the sequential change of individual biomarkers until the day of thrombotic events. The box-whisker plot indicated median and upper/lower quartile of the data of nine patients complicated with thrombosis whose respiratory conditions were ameliorated within a week. [file Image_2.TIF]
