# Supplementary material for: Arterial and Venous Thrombosis Complicated in COVID-19: A Retrospective Single Center Analysis in Japan
Source: Front Cardiovasc Med. 2021 Nov 19;8:767074. doi: 10.3389/fcvm.2021.767074 (PMC8639692; doi:10.3389/fcvm.2021.767074)
Supplement: Supplementary Table 1 — Baseline characteristics of the patients in our cohort. SD, standard deviation; COPD, chronic obstructive pulmonary disease. [file Table_1.docx]

Supplemental Table1: Baseline characteristics of the patients in our cohort

|  | **All patients** | **Mild** | **Moderate** | **Severe** |
| --- | --- | --- | --- | --- |
|  | **(n=516)** | **(n=279)** | **(n=128)** | **(n=109)** |
| **SARS-CoV-2 VOC/VOI, n (%)** | 152(30%) | 83(30%) | 39(31%) | 30(28%) |
| **Baseline characteristics** |  |  |  |  |
| Age, mean (SD) | 57.2(17.5) | 51.8(18.5) | 63.1(13.5) | 64(14.2) |
| ≧65 years, n (%) | 198(38%) | 73(26%) | 65(51%) | 60(55%) |
| Male gender, n (%) | 358(69%) | 175(63%) | 93(73%) | 90(83%) |
| Body mass index (kg/m^2^), mean (SD) | 24.3(4.9) | 23.9(5.1) | 24.2(4.0) | 25.7(5.3) |
| >30 kg/m^2^, n (%) | 53(11%) | 25(9.9%) | 9(7.6%) | 19(18%) |
| Current smoker, n (%) | 87(19%) | 49(19%) | 22(19%) | 16(19%) |
| **Comorbidities** |  |  |  |  |
| Diabetes mellitus, n (%) | 112(22%) | 46(17%) | 30(23%) | 36(33%) |
| Hypertension, n (%) | 173(34%) | 71(26%) | 50(39%) | 52(48%) |
| Hyperlipidemia, n (%) | 82(16%) | 38(14%) | 21(16%) | 23(21%) |
| COPD, n (%) | 14(2.7%) | 4(1.4%) | 8(6.3%) | 2(1.8%) |
| Asthma, n (%) | 37(7.6%) | 24(9%) | 7(5.9%) | 6(5.8%) |
| eGFR < 60ml/min/1.73m^2^, n (%) | 149(29%) | 62(22%) | 43(34%) | 44(40%) |
| History of coronary artery disease, n (%) | 19(3.7%) | 7(2.5%) | 5(3.9%) | 7(6.4%) |
| History of cerebral infarction, n (%) | 24(4.7%) | 9(3.2%) | 7(5.5%) | 8(7.3%) |
| History of malignancy, n (%) | 70(14%) | 26(9.4%) | 28(22%) | 16(15%) |
| Active malignancy, n (%) | 14(2.7%) | 4(1.4%) | 7(5.5%) | 3(2.8%) |
| Autoimmune disease, n (%) | 30(5.8%) | 19(6.8%) | 3(2.3%) | 8(7.3%) |

SD: Standard Deviation. COPD: Chronic Obstructive Pulmonary Disease.
