# Supplementary material for: Arterial and Venous Thrombosis Complicated in COVID-19: A Retrospective Single Center Analysis in Japan
Source: Front Cardiovasc Med. 2021 Nov 19;8:767074. doi: 10.3389/fcvm.2021.767074 (PMC8639692; doi:10.3389/fcvm.2021.767074)
Supplement: Supplementary Table 2 — Detail pattern of thrombosis complicated in the individual cases with COVID-19. DVT, Deep vein thrombosis; PE, Pulmonary embolism; PCI, Percutaneous coronary intervention. Distal or proximal DVT was defined as whether thromboses were identified in the calf vein or more proximal deep veins (59). In terms of severity of PE, massive, sub-massive, or non-massive was defined as whether PE with sustained hypotension, PE with normotension but with either right ventricular dysfunction and myocardial necrosis, or PE with the absence of clinical markers of adverse prognosis that define massive or sub-massive PE (60). [file Table_2.docx]

Supplemental Table 2: Detail pattern of thrombosis complicated in the individual cases with COVID-19

| COVID-19 patients with thrombosis | Number of cases | Severity of COVID-19 at the thrombotic events | | | Clinical significances of thrombotic events | | |
| --- | --- | --- | --- | --- | --- | --- | --- |
|  |  | mild | moderate | Severe | Deterioration of respiratory condition | Escalation of anticoagulation/antiplatelet treatment | Additional interventions |
| Arterial thrombosis |  |  |  |  |  |  |  |
| Acute coronary syndrome | 6 | 2 | 0 | 4 | 2 | 3 | 1 case received PCI |
| Acute coronary syndrome and intracardiac thrombosis | 2 | 1 | 0 | 1 | 1 | 2 | 1 case received PCI |
| Aortic thrombosis and arterial obstruction of the upper extremity | 1 | 1 | 0 | 0 | 0 | 1 |  |
| Cerebral infarction | 3 | 0 | 2 | 1 | 1 | 2 |  |
| Cerebral infarction, aortic thrombosis, and splenic infarction | 1 | 0 | 1 | 0 | 1 | 1 | 1 case received thrombectomy |
| Renal and splenic infarction | 1 | 0 | 1 | 0 | 1 | 1 |  |
| Venous thrombosis ^†^ |  |  |  |  |  |  |  |
| Proximal DVT | 3 | 0 | 0 | 3 | 1 | 1 |  |
| Non-massive PE | 11 | 2 | 4 | 5 | 6 | 6 |  |
| Proximal DVT and sub-massive PE | 1 | 0 | 1 | 0 | 0 | 1 |  |
| Proximal DVT and non-massive PE | 1 | 0 | 1 | 0 | 1 | 1 |  |
| Distal DVT and sub-massive PE | 1 | 0 | 0 | 1 | 0 | 1 |  |
| Distal DVT and non-massive PE | 1 | 1 | 0 | 0 | 0 | 1 |  |
| Total | 32 | 7 | 10 | 15 | 14 | 21 |  |

DVT: Deep vein thrombosis. PE: Pulmonary embolism. PCI: Percutaneous coronary intervention.

^†^Distal or proximal DVT was defined as whether thromboses were identified in the calf vein or more proximal deep veins [59]. In terms of severity of PE, massive, sub-massive, or non-massive was defined as whether PE with sustained hypotension, PE with normotension but with either right ventricular dysfunction and myocardial necrosis, or PE with the absence of clinical markers of adverse prognosis that define massive or sub-massive PE [60].
